# Protective effect of 1α,25-dihydroxyvitamin D3 on effector CD4^+^ T cell induced injury in human renal proximal tubular epithelial cells

**DOI:** 10.1371/journal.pone.0172536

**Published:** 2017-02-28

**Authors:** Byung Ha Chung, Bo-Mi Kim, Kyoung Chan Doh, Mi-La Cho, Kyoung Woon Kim, Chul Woo Yang

**Affiliations:** 1 Convergent Research Consortium for Immunologic Disease, The Catholic University of Korea Seoul, Korea; 2 Transplant Research Center, Seoul St. Mary's Hospital, College of Medicine, The Catholic University of Korea, Seoul, Korea; 3 Division of Nephrology, Department of Internal Medicine, Seoul St. Mary's Hospital, College of Medicine, The Catholic University of Korea, Seoul, Korea; University of Florida College of Medicine, UNITED STATES

## Abstract

**Background:**

The aim of this study was to investigate the protective effect of 1α,25-dihydroxyvitamin D3 [1,25(OH)_2_D3] on effector CD4^+^ T cells or on inflammatory cytokine-induced injury in human renal proximal tubular epithelial cells (HRPTEpiC).

**Methods:**

First, we investigated the effect of 1,25(OH)_2_D3 on CD4^+^ T cell proliferation. Second, we examined the effect of 1,25(OH)_2_D3 on inflammatory cytokine secretion or fibrosis in HRPTEpiC induced by inflammatory cytokines or activated CD4^+^ T cells using ELISA and real-time PCR. Lastly, we compared urine inflammatory-cytokine (IL-6, IL-8) or KIM-1 levels in kidney transplant recipients low serum 25-hydroxyvitamin D (25(OH)D) group (< 20 ng/mL) (n = 40) and normal 25(OH)D group (n = 50).

**Results:**

Pre-incubation with 1,25(OH)_2_D3 significantly reduced the percentages of Th1 and Th17 cells compared to that of Th0 condition (P < 0.05 for each). In contrast, 1,25(OH)_2_D3 increased the proportion of Th2 and Treg cells in a dose-dependent manner (P < 0.05 for each). Treatment of HRPTEpiC with inflammatory cytokines (TNF-α, IL-17, and TGF-β) or effector CD4^+^ T cells resulted in increased production of IL-6, IL-8, or KIM-1 from HRPTEpiC in a dose-dependent manner. However, treatment with 1,25(OH)_2_D3 significantly reduced the level of these cytokines (P < 0.05 for all). Western blot analysis demonstrated that the mTOR/STAT3/ERK pathway was downregulated by 1,25(OH)_2_D3 in HRPTEpiC. Furthermore, the concentrations of urine IL-6/creatinine (P < 0.05) and Kim-1/creatinine (P < 0.05) were higher in the low 25(OH)D group than in the normal 25(OH)D group in kidney transplant recipients.

**Conclusion:**

The results of this study suggests that vitamin D may have a significant role in the regulation of inflammation in allograft tissue in kidney transplant recipients.

**Trial registration:**

All participants provided written informed consent in accordance with the Declaration of Helsinki. This study was approved by the Institutional Review Board of Seoul St. Mary’s Hospital (KC13TNMI0701).

## Introduction

Recent studies have demonstrated the modulatory effects of vitamin D on various immune cells [1, 2]. Studies also demonstrate its significant association with immune disorders [3–7]. Low serum levels of 25-hydroxyvitamin D (25(OH)D) are frequently associated with autoimmune diseases or graft versus host disease after hematopoietic stem cell transplantation. [3, 8, 9] Treatment with 1α,25 dihydroxyvitamin D3 (1,25(OH)_2_D3) was shown to have significant therapeutic effects on those disorders. [10, 11] In kidney transplantation, 25(OH)D insufficiency was also associated with high incidence of acute rejection or the development of urinary tract infection. This may be owing to the modulatory effect of vitamin D on immune cells. [12–14]

Previous studies have mainly focused on the effect of 1,25(OH)_2_D3 on the proliferation or activation of immune cells. However, the effect of 1,25(OH)_2_D3 on the target tissue or immune cell has not been extensively studied. [3–7] For example, the effect of 1,25(OH)_2_D3 on allograft tissue, which is the target of alloimmune effector CD4^+^ T cells or inflammatory cytokines in acute rejection, has not been fully investigated. The rejection process is not only the activation of alloimmune effector T cells, but also the injury to allograft kidney tissues. Therefore, the direct protective effect of 1,25(OH)_2_D3 on allograft tissue against immune cells or cytokines needs to be elucidated for establishing the preventive effect of 1,25(OH)_2_D3 on acute rejection.

Accordingly, we evaluated the effect of 1,25(OH)_2_D3 treatment on effector CD4^+^ T cell proliferation as well as on allograft tissue injury induced by T cells. To address this, we employed an *in vitro* experimental set up using human renal proximal tubular epithelial cell lines (HRPTEpiC) and human CD4^+^ T cells. Additionally, we examined the association between serum 25-hydroxyvitamin D (25(OH)D) levels and urine inflammatory cytokine levels or tubule injury markers in kidney transplant recipients.

## Materials and methods

### Patient populations and study design

To investigate the protective effect of 1,25(OH)_2_D3 on HRPTEpiC from activated CD4^+^ T cells or inflammatory cytokines, we designed three separate experiments, two *in vitro* analyses and an *ex vivo* analysis. First, we investigated the suppressive effect of 1,25(OH)_2_D3 on CD4^+^ T cell proliferation. We enrolled six healthy volunteers for peripheral blood donation. The age was 31.8±5.4 years and four were male and two were females. The serum 25(OH)D level at the time of blood donation was 30.3 ± 4.9 ng/mL. We investigated the effect of 1,25(OH)_2_D3 on CD4^+^ T cell proliferation by FACS analysis and ELISA.

Second, we investigated the effect of 1,25(OH)_2_D3 on inflammation or fibrosis induced by inflammatory cytokines or activated CD4^+^T cells in HRPTEpiC, using ELISA and real-time PCR. Further, we used western blotting to analyze mTOR/STAT3 signaling as a potential mechanism by which 1,25(OH)_2_D3 exerts its effect on HRPTEpiC.

Third, we collected serum and urine samples from 90 kidney-transplant recipients with stable allograft function (Table 1). We measured serum 25-hydroxyvitamin D (25(OH)D) levels and divided them into normal 25(OH)D group (25(OH)D ≥ 20 ng/mL) and low 25(OH)D group (25(OH)D < 20 ng/mL). We also measured urine IL-6, IL-8, KIM-1, and creatinine levels in each subject. We compared urine IL-6/creatinine and urine IL-8/creatinine and KIM-1/creatinine between normal 25(OH)D group and low 25(OH)D group.

**Table 1:** Baseline characteristics of the patients cohort to investigate the association between 25(OH)2D and urine cytokine level.

	Normal 25(OH)D (n = 50)	Low 25(OH)D (n = 40)	P
**Age (year)**	45.7 ± 11.4	46.7 ± 11.2	0.69
**Male, n (%)**	27 (54)	22 (55)	1.0
**Primary renal disease**			0.82
Diabetes Mellitus (n, %)	14 (28)	11 (28)	
Chronic glomerulonephritis (n, %)	15 (30)	10 (25)	
Hypertension (n, %)	7 (14)	8 (20)	
ADPKD (n, %)	2 (4)	1 (3)	
SLE (n, %)	1 (2)	0 (0)	
Others (n, %)	1 (2)	2 (5)	
Unknown (n, %)	10 (20)	8 (20)	
**Post-transplant month**	67.2 ± 83.3	74.9 ± 79.7	0.67
**MDRD eGFR (mL/min/1.73 m**^**2**^**)**	45.5 ± 20.4	44.0 ± 20.7	0.73
**25(OH)D (ng/mL)**	28.1 ± 7.5	12.9 ± 4.5	
**HLA mismatch**	3.0 ± 1.7	2.9 ± 1.4	0.81
**Donor type, n (%)**			
LRD, n (%)	23 (46)	22 (55)	
LURD, n (%)	11 (22)	9 (23)	0.58
DD, n (%)	16 (32)	9 (23)	
**Main Immune suppressant**			
Tac, n (%)	38 (76)	29 (73)	0.65
CsA, n (%)	10 (20)	7 (18)	
mTOR inhibitor, n (%)	1 (2)	3 (8)	
AZA, n (%)	1 (2)	1 (3)	
**Induction therapy**			
Anti-thymocyte globulin (n, %)	11 (22)	4 (10)	0.16
Basliiximab (n, %)	39 (78)	36 (90)	

25(OH)2D, 25-hydroxyvitamin D; ADPKD, autosomal dominant polycystic kidney disease; SLE, systemic lupus erythematosus; MDRD, Modification of diet in renal disease; eGFR, estimated glomerular filtration rate; LRD, living related donor; LURD, living unrelated donor; DD, deceased donor; Tac, tacrolimus; MMF, mycophenolate mofetil; CsA, cyclosporin; mTOR, mammalian target of rapamycin; AZA, azathioprine

All participants provided written informed consent in accordance with the Declaration of Helsinki. This study was approved by the Institutional Review Board of Seoul St. Mary’s Hospital (KC13TNMI0701).

### Reagents

Recombinant human IFN-γ, IL-17, IL-22, IL-23, TNF-α, IL-6 and IL-8 were purchased from R&D Systems (Minneapolis, MN). Anti-human IFN-γ, anti-IL-17, anti- IL-22, anti-IL-23, anti-IL-6, anti-IL-8 and anti-KIM-1 antibodies were purchased from R&D Systems. Anti-CD3 and anti-CD28 were obtained from BD Biosciences (San Diego, CA), and 1,25(OH)_2_D3 was obtained from Sigma (St. Louis, MO).

### Identification of Peripheral Blood Mononuclear Cells (PBMCs)

PBMCs were prepared from heparinized blood by Ficoll–Hypaque (GE Healthcare; PA) density-gradient centrifugation. Cells were cultured as previously described [8].

Briefly, a cell suspension of 10^6^ cells/mL was prepared in RPMI-1640 medium supplemented with 10% fetal calf serum, 100 U/mL penicillin, 100 mg/mL streptomycin, and 2 mM L-glutamine. A 1-mL aliquot of the suspension was dispensed into 24-well plates (Nunc; Roskilde, Denmark), and incubated. For cytokine detection at the single-cell level, PBMCs were stimulated with 50 ng/mL phorbol myristate acetate (PMA) and 1 μg/mL ionomycin in the presence of GolgiStop (BD Biosciences, San Diego, CA) for 4 hours in 37°C.

### Induction of Th0 polarizing conditions in PBMCs

PBMC cells (5 × 10^5^) isolated from healthy individuals were incubated under appropriate conditions for 48 hours. To induce Th0 polarizing conditions, anti-CD3 (1 μg/mL) and anti-CD28 (1 μg/mL) were used. To examine the immunosuppressive effects of 1,25(OH)_2_D3, PBMCs were pre-incubated for 1 hour with 1,25(OH)_2_D3 (1, 10, and 100 nM), and stimulated as described above.

### Flow cytometry analysis for cytokine analysis in PBMCs

Flow cytometry analysis was performed on *in vitro* samples within a few hours after peripheral blood collection. The cells were surface-stained with different combinations of the following mAbs: CD4-PE/Cy7 (RPA-T4, IgG1; BioLegend, San Diego, CA) and CD25-APC (M-A251, IgG1, κ; Pharmingen). For intracellular staining, the cells were washed, fixed, permeabilized, and incubated with mAbs against IL-17 (PE, eBio64dec17, IgG1, κ; eBioscience, San Diego, CA), IFN-γ (FITC, 4S.B3, IgG1, κ; eBioscience; and PE, B27, IgG1, κ; Pharmingen), IL-4 (APC, MP4-25D2, IgG1, κ; eBioscience), and Foxp3 (FITC, PCH101, IgG2a, κ; eBioscience). Appropriate isotype controls were used for gating. Cells were analyzed using FACS Calibur flow cytometer (BD Biosciences). The data were analyzed using FlowJo software (Tree Star, Ashland, OR, USA).

### Human Renal Proximal Tubular Epithelial Cell (HRPTEpiC) line culture and experiments

The HRPTEpiC line was purchased from ScienCell Research Laboratories (ScienCell, CA, USA), and was maintained in epithelial cell medium (EpiCM, ScienCell Research Laboratories) supplemented with 2% fetal bovine serum (FBS) at 37°C and 5% CO_2_. HRPTEpiC were seeded in 24-well plates at a density of 2 × 10^5^ HRPTEpiC/mL. After one day, the medium was replaced with HRPTEpiC culture medium with or without cytokines. HRPTEpiC were stimulated with 1, 10, 50, or 100 ng/mL human recombinant IL-17 (R&D Systems, Inc. Minneapolis, MN) and/or 1, 10, or 50 ng/mL human recombinant TNF-α (R&D Systems) for 72 hours. To examine the immunosuppressive effects of 1,25(OH)_2_D3 (Sigma), HRPTEpiC were pre-incubated for 1 hour with 1,25(OH)_2_D3 (10 nM), and then stimulated as described above. The in vitro concentrations of 1,25(OH)_2_D3 were selected according to the previous studies [15–17]. Supernatants were harvested and stored at -80°C until analysis.

### Coculture of Human Renal Proximal Tubular Epithelial Cell (HRPTEpiC) line and activated CD4+T cells

For co-culture experiments with CD4^+^ T cells, HRPTEpiC were seeded in 24-well plates at 2 × 10^4^ cells/well with 1 mL of medium, and pre-incubated for 1 hour with 1,25(OH)_2_D3 (10 nM). CD4^+^ T cells (2 × 10^5^ cells/well) with or without stimulation under Th0 polarizing conditions were added to the HRPTEpiC monolayers, and the culture plates were incubated for 48 hours. The culture supernatants were collected and stored at -80°C until assayed. All cultures were set up in triplicate.

### Enzyme-Linked Immunosorbent Assay (ELISA) for cytokine analysis

The levels of cytokines such as IL-6, IL-8, and Kim-1 in urine isolated from kidney transplant recipients or IFN-r, IL-17, IL-22, IL-23, IL-6 and IL-8 in the culture supernatants from PBMCs were measured by sandwich ELISA (R&D Systems) according to the manufacturer’s instructions. Absorbance at 405 nm was measured using an ELISA microplate reader (Molecular Devices).

### Expression of Kim-1 and FN-1 mRNA by real-time reverse transcription Polymerase Chain Reaction (real-time PCR)

mRNA was extracted from in vitro PBMCs samples using the TRIzol Reagent (Molecular Research Center, Inc., Cincinnati, OH), according to the manufacturer’s instructions. cDNA was synthesized in a PerkinElmer Cetus DNA thermal cycler (PerkinElmer, Inc., Waltham, MA) using the SuperScript Reverse Transcription system (Takara, Shiga, Japan). A LightCycler 2.0 instrument (Roche Diagnostics; software version 4.0) was used for PCR amplification. All PCR reactions were performed using LightCycler FastStart DNA Master SYBR Green I (Takara), according to the manufacturer’s instructions. S2 Table shows the sense and antisense primers used for each molecule (5′→3′).

### Western blot analysis of the HRPTEpiC line

The cells were pre-incubated for 1 hour in the presence of 1,25(OH)_**2**_D3 (10 nM) and stimulated under recombinant IL-17 for another hour. The membrane was then incubated overnight at 4°C with primary antibodies against the following targets: VDR, phosphorylated mTOR, mTOR, phosphorylated Akt, Akt, phosphorylated s6k, phosphorylated STAT3 (705), STAT3, phosphorylated ERK, ERK, REDD1 (all antibodies were from Cell Signaling Technology Inc., Danvers, MA), and β-actin (Sigma). After washing in TBST (Tris-Buffered Saline and Tween 20), the reactive bands were visualized using an ECL detection kit and Hyperfilm-ECL reagents (Amersham Pharmacia, Piscataway, NJ).

### Statistical analysis

Statistical analysis was performed using SPSS software (version 16.0; SPSS Inc., Chicago, IL). Continuous variables were summarized as mean ± SD. Categorical variables were summarized as a percentage of the group total. A non-parametric, Wilcoxon signed-rank test was used to compare T cell suppression, cytokine production, and gene expression between the control and treatment groups. All data included in the *ex vivo* study were normally distributed. An independent *t -*test was used to compare urine cytokine levels between the low 25(OH)D and normal 25(OH)D groups. A *p* value of < 0.05 was considered statistically significant.

## Results

### Suppressive effect of 1,25(OH)_2_D3 on the proliferation of T cells isolated from healthy donors and cultured under Th0 polarizing conditions

**Fig 1:**
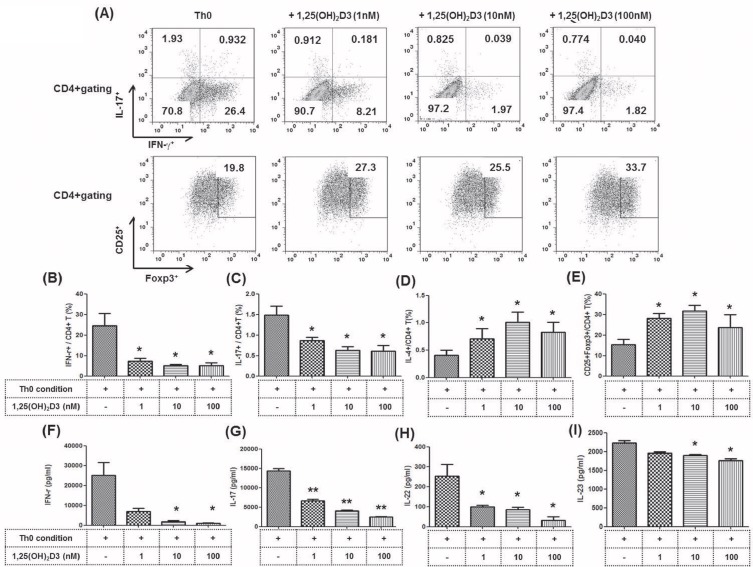
Effect of 1,25(OH)_2_D3 on CD4^+^ T cells isolated from the PBMCs of healthy donors and cultured under Th0-polarizing conditions. Human PBMCs (n = 3) were isolated from healthy subjects and pre-incubated with 1,25(OH)_2_D3 (1, 10, 100 nM) as indicated. They were then cultured under Th0-polarizing conditions (anti-CD3, 1 μg/mL and anti-CD28, 1 μg/mL) for 48 h. **(A)** PBMCs were stained with anti-CD4 PE-cy7, anti-CD25 APC, anti-IFN-γ FITC, anti-IL-17 PE, anti-IL-4 APC and anti-Foxp3 FITC. CD4^+^ T cells were gated for further analysis. Then, the percentage of **(B)** IFN-γ^+^/CD4^+^ T cells, **(C)** IL-17^+^/CD4^+^ T cells, **(D)** IL-4^+^/CD4^+^ T cells, and **(E)** CD25^+^FOXP3^+^/CD4^+^ T cells was measured by flow cytometry. The production of **(F)** IFN-γ, **(G)** IL-17, **(H)** IL-22 and **(I)** IL-23 by Th0-polarizing CD4^+^T cells and secretion into the culture supernatant by ELISA. Bars represent the mean 0± SD. * P <0.05, ** P <0.01 vs. Th0 condition. 1,25(OH)_2_D3, 1α,25 dihydroxy-vitamin D3; PBMC, peripheral blood mononuclear cells.

We performed an *in vitro* study to investigate the impact of 1,25(OH)_2_D3 on the proliferation of various CD4^+^ T cell subsets. PBMCs were isolated from 6 healthy individuals and cultured under Th0-polarizing conditions. The proportions of Th1 (CD4^+^ IFN-γ^+^), Th2 (CD4^+^ IL-4^+^), Th17 (CD4^+^ IL-17^+^), and Treg (CD4^+^ FOXP3^+^) cells out of the total CD4^+^ T cell population are presented in Fig 1A. Pre-incubation with 1,25(OH)_2_D3 (1, 10, and 100 nM) significantly reduced the percentage of Th1 and Th17 cells compared to that of Th0 condition (*P* < 0.05 for the Th0 condition) (Fig 1B and 1C and S1 Table). In contrast, 1,25(OH)_2_D3 increased the proportion of Th2 and Treg cells in a dose-dependent manner (*P* < 0.05 for the Th0 condition) (Fig 1D and 1E and S1 Table). In the culture medium, pre-incubation with 1,25(OH)_2_D3 significantly reduced the production of Th1 (IFN-γ) and Th17 cytokines (IL-17, IL-22, IL-23) compared to that of Th0, following a pattern similar to that of cellular expression analyzed by FACS (*P < 0.05, **P < 0.01 for the Th0 condition) (Fig 1F–1I).

### Protective effect of 1,25(OH)_2_D3 on inflammatory cytokine-induced inflammation in HRPTEpiC

**Fig 2:**
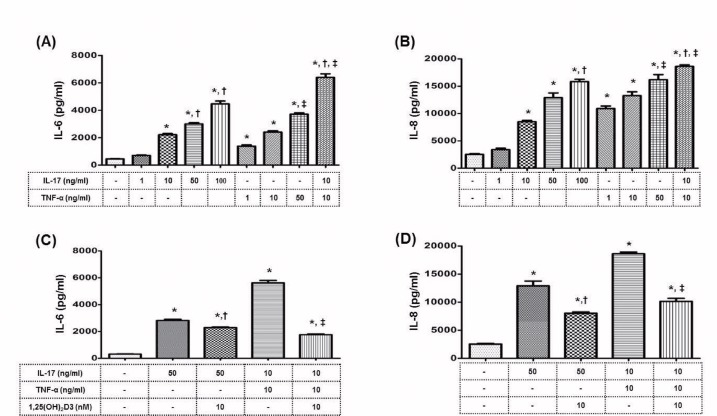
Effect of 1,25(OH)_2_D3 on the production of inflammatory cytokines by HRPTEpiC, induced by recombinant human IL-17 (rhIL-17) or human TNF-α. HRPTEpiC were cultured with rhIL-17 (0, 1, 10, 50, or 100 ng/mL) or TNF-α (0, 1, 10, or 50 ng/mL) for 48 hours, and the production of **(A)** IL-6 and **(B)** IL-8 was measured (n = 3). Note that IL-6 and IL-8 levels were significantly increased by IL-17 or TNF-α in a dose-dependent manner. *P<0.05 vs. Nil and ^†^P<0.05 vs. IL-17 1 ng/mL and ^‡^P<0.05 vs. TNF-α 1 ng/mL. **(C)** IL-6 and **(D)** IL-8 production by HRPTEpiC which were pretreated with 1,25(OH)_2_D3 (10 nM) as indicated, and then cultured for 48 hours with IL-17 (0, 10, or 50 ng/mL) or TNF-α (10 ng/mL). Note that the addition of 1,25(OH)_2_D3 significantly decreased the IL-6 or IL-8 level, which was increased by IL-17 or TNF-α. *P<0.05 vs. Nil and ^†^P<0.05 vs. IL-17 50ng/mL and ^‡^P<0.05 vs. IL-17 10ng/mL + TNF-α 10 ng/mL.

To evaluate the direct protective effect of 1,25(OH)_2_D3 on the target organ, we investigated whether 1,25(OH)_2_D3 protects HRPTEpiC against Th17 cytokine- or activated CD4^+^ T cell-induced inflammation. HRPTEpiC were cultured with IL-17 or TNF-α, and IL-6 and IL-8 levels were measured after 72-hour incubation. Fig 2A and 2B show that IL-17 and TNF-α induced IL-6 and IL-8 production in a dose-dependent manner. Treatment with 1,25(OH)_2_D3 significantly reduced the levels of these cytokines (*P* < 0.05 for all) (Fig 2C and 2D and S2 Table).

### The effect of 1,25(OH)_2_D3 on Kidney Injury Marker (KIM-1) and fibronectin 1 in HRPTEpiC

**Fig 3:**
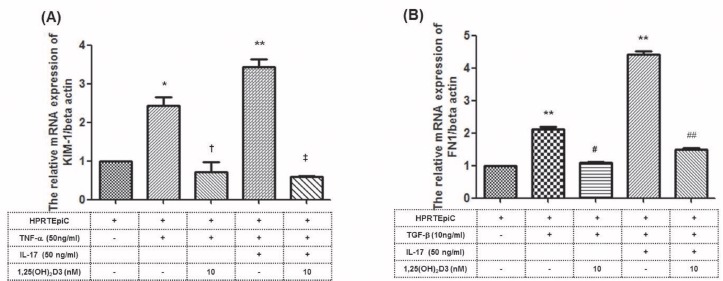
Effect of 1,25(OH)_2_D3 on the production of KIM-1 and fibronectin 1 from HRPTEpiC induced by recombinant human IL-17 (rhIL-17), human TNF- α or TGF-beta. **(A)** The expression of KIM-1 by HRPTEpiC was pretreated with 1,25(OH)_2_D3 (10 nM) as indicated, and then cultured for 24 hours with TNF-α (50 ng/mL) and/or IL-17 (50ng/mL) (n = 3). The expression of KIM-1 was measured by real-time PCR. Note that addition of 1,25(OH)_2_D3 significantly decrease KIM-1 expression which was increased by TNF-α and IL-17. *P<0.05, **P<0.01 vs. Nill and ^†^P<0.05 vs. TNF- α 50 and ^‡^P<0.05 vs. TNF-α+IL-17. **(B)** The expression of Fibronectin-1 by HRPTEpiC was pretreated with 1,25(OH)_2_D3 (10 nM) as indicated, and then cultured for 24 hours with TGF-β (10 ng/mL) and/or IL-17 (50ng/mL) (n = 3). The expression of fibronectin-1 was measured by real-time PCR. 1,25(OH)_2_D3 significantly decreased fibronectin-1 expression, which was increased by TGF-β (10 ng/mL) and IL-17. **P<0.01 vs. Nill and ^#^P<0.05 vs. TGF- β 10 and ^##^P<0.05 vs. TGF- β+IL-17.

We performed real-time PCR analyses to evaluate the effect of 1,25(OH)_2_D3 on KIM-1 mediated by IL-17 as well as TNF-α, and fibronectin 1 mediated by TGF-β as well as IL-17 in HRPTEpiC. HRPTEpiC were cultured with TNF-α and IL-17, and KIM-1 expression was measured after 24-hour incubation. Fig 3A and S3 Table shows that TNF-α and IL-17 induced KIM-1 expression. However, treatment with 1,25(OH)_2_D3 significantly reduced the expression of KIM-1 (*P < 0.05, **P < 0.01 vs. Nil; ^**†**^*P* < 0.05 vs. TNF-α; and ^**‡**^*P* < 0.05 vs. TNF-α + IL-17). Fig 3B and S3 Table shows that TGF-β and IL-17 induced fibronectin-1 expression. Treatment with 1,25(OH)_2_D3 significantly reduced the expression of fibronectin 1 (**P < 0.01 vs. Nil; ^#^*P* < 0.05 vs. TGF-b; and ^##^*P* < 0.01 vs. TGF-b+IL-17)

### Protective effect of 1,25(OH)_2_D3 on activated CD4^+^ T cell- induced inflammation in HRPTEpiC

**Fig 4:**
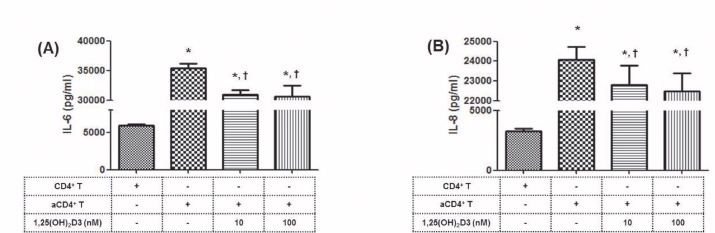
Effect of 1,25(OH)_2_D3 on the production of inflammatory cytokines by HRPTEpiC, induced by activated CD4^+^ T cells. **(A)** IL-6 and **(B)** IL-8 production by HRPTEpiC which were pre-treated with 1,25(OH)_2_D3 (10 nM) as indicated, and then co-cultured for 48 hours with activated CD4+T cells. Note that treatment with 1,25(OH)_2_D3 did suppress the production of IL-6 and IL-8 by HRPTEpiC. *P<0.05 vs. CD4^+^ T control and ^†^P<0.05 vs. activated CD4^+^ T. Values are expressed as the mean and SD of triplicate cultures. 1,25(OH)_2_D3, 1α,25 dihydroxy-vitamin D3; HRPTEpiC, human renal proximal tubular epithelial cells

We co-cultured HRPTEpiC with CD4^+^ T cells with or without stimulation under Th0 conditions. Co-culturing HRPTEpiC with CD4^+^ T cells without stimulation did not increase the production of IL-6 or IL-8. However, co-culturing with activated CD4^+^ T cells under Th0 conditions significantly increased HRPTEpiC production of IL-6 and IL-8 compared to the unstimulated co-culture with CD4^+^ T cells (P < 0.05). However, addition of 1,25(OH)_2_D3 significantly decreased the HRPTEpiC production of these cytokines compared to that by activated CD4^+^ T cells (*P < 0.05 vs. CD4^+^ T control, and ^**†**^*P* < 0.05 vs. activated CD4^+^ T cells) (Fig 4A and 4B and S4 Table).

### Pathways involved in the protective effect of 1,25(OH)_2_D3 on HRPTEpiC

**Fig 5:**
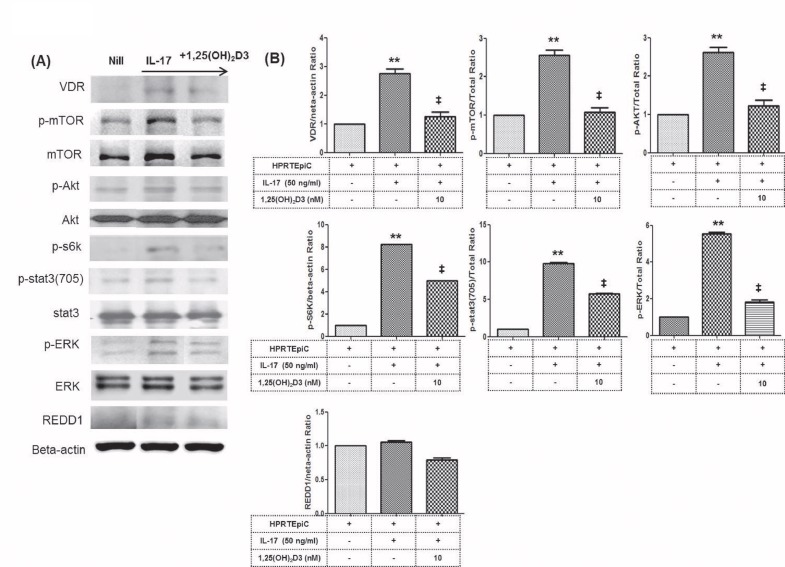
Effects of 1,25(OH)_2_D3 on the expression of mTOR and STAT3 proteins in HRPTEpiC. **(A)** Immunoblotting of VDR, *p*-mTOR, mTOR, *p-*Akt, Akt, *p-*s6k, *p-*STAT3(705), STAT3, *p*-ERK, ERK and REDD1 in HRPTEpiC pretreated with or without 1,25(OH)_2_D3 (10 nM) and then cultured with recombinant IL-17 for 1 hour. **(B)** Stimulation of HRPTEpiC with recombinant IL-17 activated the phosphorylation of mTOR, Akt, STAT3,ERK and s6k as detected by Western blotting and shown by the ratio of phosphorylated to total proteins. Note that combined use of 1,25(OH)_2_D3 resulted in the most inhibitory effect on the expression of VDR, mTOR, Akt, STAT3, ERK and s6k. Bars show the mean ±SD results in 3 patients, in 1 of 3 independent experiments. **P<0.01 vs. Nill and ^‡^P<0.01 vs. IL-17 1,25(OH)_2_D3, 1α,25 dihydroxy-vitamin D3

Using the HRPTEpiC cell line, we investigated the molecular mechanisms through which 1,25(OH)_2_D3 modulates inflammatory cytokines. As shown in Fig 5 and S1 Fig, the levels of phosphorylated mTOR, STAT3 (705), AKT (pAKT), s6k and ERK showed significant increases in IL-17 compared to the nil condition. Treatment with 1,25(OH)_2_D3 significantly decreased the levels of phosphorylated mTOR, STAT3 (705), AKT(pAKT), p70S6k, and its downstream protein ERK compared to IL-17 (*P* < 0.05 for each) (Fig 5A and 5B and S1 Fig).

### Association between serum 25(OH)D levels and urine IL-6, IL-8, KIM-1, and creatinine levels in urine from kidney transplant recipients

**Fig 6:**
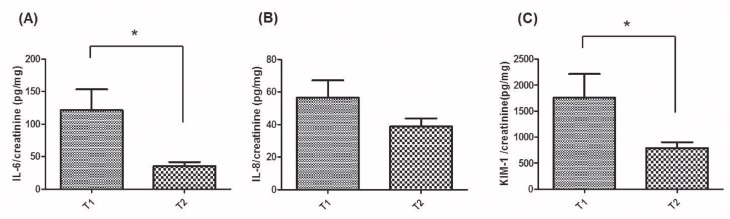
1,25(OH)_2_D3 and urine inflammatory cytokine level in urine from kidney transplant recipients. The production of **(A)** *IL-6/creatinine*, **(B)** *IL-8/creatinine* **(C)** *KIM-1/creatinine* was measured using ELISA in urine from kidney transplant recipients. We divided patients group into two groups based on serum 25(OH)D level; Low 25(OH)D group (25(OH)D <20 ng/mL, T1 (n = 40)), Normal 25(OD)D group (25(OH)D > 20 ng/mL, T2 (n = 50)) from kidney transplant recipients. Bars show the means. * *P*<0.05 vs. T1.

To avoid dilution and to standardize the samples, urinary levels of IL-6, IL-8, and KIM-1 were expressed as the ratio of cytokine to urinary creatinine (pg/mg). The concentration of urine IL-6/creatinine (*p* < 0.05) and urine IL-8/creatinine was higher in the low 25(OH)D group (T1; n = 40) than in the normal 25(OH)D group (T2; n = 50) from kidney transplant recipients (Fig 6A and 6B and S5 Table). Furthermore, the concentration of urine KIM-1/creatinine (*p* < 0.05) was also higher in the low 25(OH)D group than in the normal 25(OH)D group (Fig 6C and S5 Table). There was an inverse relationship between serum 25(OH)D and urine IL-6/creatinine (*p* < 0.05) as well as between serum 25(OH)D and KIM-1/creatinine (*p* < 0.05) in kidney transplant recipients.

## Discussion

In this study, we intended to find the direct protective effect of 1,25(OH)_2_D3 on HRPTEpiC against effector T cells or cytokines. We observed that pre-treatment with 1,25(OH)_2_D3 effectively regulated not only the proliferation of effector T cells but also suppressed injury induced by activated T cells or inflammatory cytokines, which may represent allograft rejection. Tubular epithelial cells are a key feature of acute graft rejection as well as of any inflammatory reaction in transplanted kidneys [18–20].

In this regard, we performed *in vitro* analyses to investigate the effect of 1,25(OH)_2_D3 on the proliferation of effector CD4^+^ T cells as well as the direct protective effect on HRPTEpiC from effector T cell-induced injury. Our *in vitro* study using human PBMCs pre-incubated with 1,25(OH)_2_D3 demonstrated significant reduction in the percentage of Th1 and Th17 cells compared to that of Th0 condition (*P* < 0.05 for each). In contrast, 1,25(OH)_2_D3 increased the proportion of Th2 and Treg cells in a dose-dependent manner (*P* < 0.05 for each). These observations correlate with those from previous studies showing that 1,25(OH)_2_D3 suppresses T cell proliferation, [21] and results in a shift from a Th1 to a Th2 phenotype [22, 23]. 1,25(OH)_2_D3 has also been shown to affect T cell maturation with a skewing away from the inflammatory Th17 phenotype, [24, 25] as well as to facilitate the induction of T regulatory cells [25–28].

Further, we evaluated the direct protective effect of 1,25(OH)_2_D3 on activated T cell-induced target organ injury, which may be similar to allograft rejection.

Most of kidney diseases is associated with acute tubular cell injury and dysfunction [29, 30]. Renal tubular epithelial cells play a crucial role in renal function. Renal tubular epithelial cells can produce inflammatory mediators such as cytokines (IL-1, TNF-α, IL-17) and chemokines and actively participate in acute inflammatory processes by affecting and directing leukocyte chemotaxis via the production of IL-8 [19, 31–35].

Based on the above background, we designed two separate experiments using HRPTEpiC: first, pre-treatment with inflammatory cytokines; second, co-culture with activated T cells. In response to the treatment of HRPTEpiC with inflammatory cytokines such as TNF-α, IL-17 induced the production of IL-6, IL-8, and KIM-1, whereas TGF-β increased the expression of fibronectin-1 in a dose-dependent manner. However, treatment with 1,25(OH)_2_D3 significantly reduced the levels of these cytokines or molecules (*P* < 0.05 for all). In the second experiment, we investigated whether 1,25(OH)_2_D3 protects HRPTEpiC against activated CD4^+^ T cell-induced inflammation. Our results showed that activated CD4^+^T cell-induced inflammation also induced IL-6 and IL-8 production from HRPTEpiC. Similarly, treatment with 1,25(OH)_2_D3 significantly reduced the levels of these cytokines (*P* < 0.05 for all).

Next, we investigated the molecular signaling pathway involved in the suppressive effects of 1,25(OH)_2_D3 on HRPTEpiC. We focused on the mTOR/Akt pathway, which plays an essential role in the regulation of kidney inflammation and fibrosis. [36–40]. We also investigated the activity of Akt and p70S6k, which are located either upstream or downstream of the mTOR/STAT3 pathway, and are significantly associated with the activity of mTOR [41, 42]. Activation of mTOR most prominently results in the phosphorylation of two downstream targets, ribosomal S6 Kinase (S6K) and eukaryotic translation-initiation factor 4E-binding protein (4E-BP), which stimulate ribosome biogenesis and translation in order to increase cell mass [43, 44].

Our results showed that 1,25(OH)_2_D3 has the potential to suppress mTOR signaling by reducing the protein expression of mTOR, STAT3, ERK and reducing p-p70S6 kinase activity in HRPTEpiC (Fig 5A and 5B and S1 Fig). These findings indicate the therapeutic potential of 1,25(OH)_2_D3 in HRPTEpiC.

Finally, we investigated the association between serum 25(OH)D levels and urine IL-6, IL-8, KIM-1, and creatinine levels in kidney transplant recipients with stable allograft function. In the clinical setting, the measurement of urinary IL-6, IL-8, and KIM-1 levels is thought to be a potential biomarker of the localization and severity of inflammation within the renal tubular system [45, 46]. In this *ex vivo* study, we used serum 25(OH)D levels instead of 1,25(OH)_2_D3 levels because it shows some advantages. First, 25(OH)D is easy to measure and hence is widely used for the diagnosis of vitamin D deficiency in the clinical practice. Second, it better represents the immune status of patients and also, its level is not affected by renal function [47, 48]. In addition, 25(OH)D level shows very significant correlation with 1,25(OH)2D3 level [49]. As a result, an inverse relationship between serum 25(OH)D and urine IL-6/creatinine (*p* < 0.05) as well as between serum 25(OH)D and KIM-1/creatinine (*p* < 0.05) in kidney transplant recipients without rejection or urinary tract infection were detected. These findings provide evidence of the advantages of sustaining normal vitamin D level in kidney transplant recipients.

However, this study has some limitations. First, the study only evaluated T cell mediated rejection in renal tubular epithelial cell, which accounts for only a small portion of allograft rejection. Additional *in vitro* studies using vascular endothelial cells, which are also important targets of alloimmune cells or alloantibodies, may be required to fully represent the entire spectrum of allograft rejection in clinical practice. Second, this study did not show the direct clinical benefit of normal 25(OH)D levels and low inflammatory cytokine expression in urine from kidney transplant recipients. However, several previous studies have already demonstrated the clinical significance of normal 25(OH)D levels for the prevention of allograft rejection or urinary tract infection. Therefore, we thought that the observations from this study might explain one of the mechanisms through which vitamin D exerts its beneficial effect. [12, 13, 50]

In conclusion, 1,25(OH)_2_D3 treatment showed significant suppressive effect on renal tubular inflammation induced by activated T cells or inflammatory cytokines via regulation of the mTOR/STAT3 pathway. In our *ex vivo* study, serum 25(OH)D showed significant association with urine inflammatory cytokine levels, which is consistent with the findings of our *in vitro* study.

The results of this study suggests that vitamin D may have an significant role in the regulation of inflammation in allograft tissue in kidney transplant recipients.

## Supporting information

S1 Fig The images of entire gel membrane in Fig 5. (PDF)

S1 Table The numerical data of Fig 1. (PDF)

S2 Table The numerical data of Fig 2. (PDF)

S3 Table The numerical data of Fig 3. (PDF)

S4 Table The numerical data of Fig 4. (PDF)

S5 Table The numerical data of Fig 6. (PDF)

## Abbreviations

1,25(OH)_2_D3: 1α,25 dihydroxy-vitamin D3
25(OH)D: 25-hydroxyvitamin D
HRPTEpiC: human renal proximal tubular epithelial cells
KTR: kidney transplant recipient
PBMC: peripheral blood mononuclear cells
Th1: T helper type 1
Th2: T helper type 2
Th17: T helper type 17
Treg: T regulatory
IL-6: Interleukin-6
IL-8: Interleukin-8
KIM-1: Kidney Injury Molecule-1
mTOR: mechanistic target of rapamycin
Stat3: Signal transducer and activator of transcription 3
