# Supplementary material for: Protective effect of 1α,25-dihydroxyvitamin D3 on effector CD4+ T cell induced injury in human renal proximal tubular epithelial cells
Source: PLoS One. 2017 Feb 28;12(2):e0172536. doi: 10.1371/journal.pone.0172536 (PMC5330482; doi:10.1371/journal.pone.0172536)
Supplement: S1 Table — (PDF) [file pone.0172536.s002.pdf]

S1 Table

IFN-r+

Th0	Th0+1,25(OH)2D3 (1uM)	Th0+1,25(OH)2D3 (10uM)	Th0+1,25(OH)2D3 (100uM)
35.9	9.17	4.31	7.77
22.3	8.07	5.52	4.49
15.4	4.47	5.78	3.12

IL-17+

Th0	Th0+1,25(OH)2D3 (1uM)	Th0+1,25(OH)2D3 (10uM)	Th0+1,25(OH)2D3 (100uM)
1.18	0.86	0.444	0.877
1.92	1.01	0.685	0.544
1.36	0.726	0.749	0.399

IL-4+

Th0	Th0+1,25(OH)2D3 (1uM)	Th0+1,25(OH)2D3 (10uM)	Th0+1,25(OH)2D3 (100uM)
0.48	0.689	0.969	0.56
0.225	0.4	0.705	0.748
0.521	1.04	1.35	1.18

CD25+Foxp3+

Th0	Th0+1,25(OH)2D3 (1uM)	Th0+1,25(OH)2D3 (10uM)	Th0+1,25(OH)2D3 (100uM)
19.5	24.8	32.6	35.2
10.4	27	26.4	22.3
15.9	32.6	36.1	13.9
