# Supplementary material for: Protective effect of 1α,25-dihydroxyvitamin D3 on effector CD4+ T cell induced injury in human renal proximal tubular epithelial cells
Source: PLoS One. 2017 Feb 28;12(2):e0172536. doi: 10.1371/journal.pone.0172536 (PMC5330482; doi:10.1371/journal.pone.0172536)
Supplement: S2 Table — (PDF) [file pone.0172536.s003.pdf]

S2 Table

IL-6 (pg/ml)

Nil	IL-17 1	IL-17 10	IL-17 50	IL-17 100	TNF-a 1	TNF-a 10	TNF-a 50	IL-17 10 +TNF-a 10
478.9	765.51	2314.77	2904.295	4784.905	1562.56	2564.115	3701.07	5989.27
411.8	629.65	2025.95	2923.145	4065.895	1309.07	2249	3533.78	6896.845
454	717.26	2294.675	3178.925	4536.52	1249.55	2410.41	3899.39	6294.76

IL-8 (pg/ml)

Nil	IL-17 1	IL-17 10	IL-17 50	IL-17 100	TNF-a 1	TNF-a 10	TNF-a 50	IL-17 10 +TNF-a 10
2783	3861.5	8902.58	14433.89	16603.01	11757.6	14541.93	18005.82	18147.79
2469	3005.3	8479.075	12822.17	15808.72	10814.2	13149.81	15609.49	18532.55
2349	3345.5	8177.99	11448.58	15115.51	10173.4	12149.47	14971.35	19146.8

IL-6 (pg/ml)

Nil	IL-17 50	IL-17 50 +1,25(OH)2D3 (10uM)	IL-17 10 +TNF-a 10	IL-17 10 +TNF-a 10 +1,25(OH)2D3 (10uM)
478.9	2904.3	2920	5989.27	2510.495
411.8	2923.1	2618.17	6896.845	2080.87
454	3178.9	2786.005	6294.76	2603.11

IL-8 (pg/ml)

Nil	IL-17 50	IL-17 50 +1,25(OH)2D3 (10uM)	IL-17 10 +TNF-a 10	IL-17 10 +TNF-a 10 +1,25(OH)2D3 (10uM)
2783	14434	8418.535	18147.79	11183.92
2469	12822	7664.235	18532.55	9406.27
2349	11449	8049.54	19146.8	9846.125
