# Supplementary material for: Protective effect of 1α,25-dihydroxyvitamin D3 on effector CD4+ T cell induced injury in human renal proximal tubular epithelial cells
Source: PLoS One. 2017 Feb 28;12(2):e0172536. doi: 10.1371/journal.pone.0172536 (PMC5330482; doi:10.1371/journal.pone.0172536)
Supplement: S3 Table — (PDF) [file pone.0172536.s004.pdf]

S3 Table

KIM-1/beta-actin

Nil	TNF-a 50	TNF-a 50+ +1,25(OH)2D3 10	TNF-a+IL-17	TNF-a + IL-17 + 1,25(OH)2D3 10
1	1.965641	0.8321987	3.59253	0.5803519
1	3.000078	0.008943158	3.668016	0.6484198
1	2.925189	0.5723621	3.041483	0.5605831

FN-1/beta-actin

Nil	TGF-b 10	TGF-b 10 +1,25(OH)2D3 10	TGF-b + IL-17	TGF-b + IL-17 + 1,25(OH)2D3 10
1	1.988481	1.121166	4.578897	1.542211
1	2.058605	1.031683	4.453686	1.520979
1	2.268387	1.090508	4.242751	1.399586
