# Supplementary material for: Protective effect of 1α,25-dihydroxyvitamin D3 on effector CD4+ T cell induced injury in human renal proximal tubular epithelial cells
Source: PLoS One. 2017 Feb 28;12(2):e0172536. doi: 10.1371/journal.pone.0172536 (PMC5330482; doi:10.1371/journal.pone.0172536)
Supplement: S4 Table — (PDF) [file pone.0172536.s005.pdf]

S4 Table

IL-6 (pg/ml)

CD4+T	aCD4+T	aCD4+T+1.25(OH)2D3 (10nM)	aCD4+T+1.25(OH)2D3 (100nM)
5702.1	33108.94	28031.41	25428.38
5741.58	33906.9	31216.1	26509.71
6406.16	35698.17	32062.14	28861.07
6249.3	36919.24	30471.37	33427.78
6021.27	35286.15	30521.71	31929.25
5700.96	37726.78	33442.68	37602.11

IL-8 (pg/ml)

CD4+T	aCD4+T	aCD4+T+1.25(OH)2D3 (10nM)	aCD4+T+1.25(OH)2D3 (100nM)
3422.76	22365.33	21413.06	20254.74
3321.3	23048.73	20132.96	20477.89
3261.03	22931.45	21056.32	21169.56
3810.99	24639.29	26146.76	25382.01
3687.27	26428.47	25099.34	24615.5
2121.26	25141.35	22958.07	23102.41
