# Supplementary material for: Protective effect of 1α,25-dihydroxyvitamin D3 on effector CD4+ T cell induced injury in human renal proximal tubular epithelial cells
Source: PLoS One. 2017 Feb 28;12(2):e0172536. doi: 10.1371/journal.pone.0172536 (PMC5330482; doi:10.1371/journal.pone.0172536)
Supplement: S5 Table — (PDF) [file pone.0172536.s006.pdf]

S5 Table

IL-6/creainine & 25 (OD)D

T1	T2
5.002033	33.05836
52.72772	28.22837
463.3972	1.539855
742.8355	23.61184
33.96276	6.678899
4.315504	60.68595
38.39103	10.28089
833.4244	0.4701327
13.37921	27.048
33.32081	217.2109
9.467462	25.31356
31.23722	10.24561
30.90297	192.1225
44.74207	0.7128713
14.79655	66.61755
27.70281	25.98613
156.8696	89.23661
9.251634	56.64474
6.674825	6.880388
123.1516	24.46073
9.79683	5.268806
13.37761	57.75186
295.25	17.3752
38.8539	59.4358
87.28658	21.18957
155.8165	25.97805
215.4258	15.18087
84.46575	8.511628
1.591408	41.06183
269.9138	5.402672
7.820442	65.15306
63.21429	7.368263
263.4359	17.45885
1.391635	42.52679
6.910725	21.29044
6.715833	2.404817
409.7214	8.212069
4.211256	167.5903
ND	13.01393
ND	50.79021

IL-8/creainine & 25 (OD)D

T1	T2
21.19106	24.27237
86.56931	14.61368
91.91096	34.0471
30.07018	77.36842
48.54255	60.17278
16.06434	34.67355
44.42308	12.04247
94.52325	11.07411
37.89888	19.07067
13.92197	64.10156
8.201193	122.5212
10.66053	13.20724
34.79909	49.67857
23.09654	40.22772
31.55172	44.70861
29.54719	22.10196
261.8478	143.192
36.5817	94.58553
12.0507	9.979526
12.47406	10.39491
35.63806	10.60037
50.00521	10.3272
44.67208	19.21012
43.6128	9.5
72.06649	131.2366
57.54585	9.126087
77.84703	185.5116
7.85192	15.80645
22.9387	21.90076
23.5442	111.3571
64.8006	35.8503
93.36539	31.2716
18.90304	88.77679
20.08196	32.28309
12.79083	10.00344
298.0258	17.97586
155.5979	105.6274
5.624796	59.1875
ND	14.3273
ND	11.16076

KIM-1/creainine & 25 (OD)D

T1	T2
933.4858	145.1245
4900.525	221.9728
2659.808	2465.648
2822.081	861.6711
100.0426	451.1896
349.5899	149.405
339.6763	58.58784
2244.483	46.01659
263.6404	2216.156
542.9586	955.7305
855.6128	570.9025
1808.121	119.3235
444.8208	126.5357
2397.831	546.1931
1055.861	962.2285
1881.288	694.4266
17149.48	802.0714
1387.631	1172.543
86.96154	417.2317
1222.229	3444.542
737.3977	392.3053
815.4172	252.6617
4223.635	53.7688
646.7922	3467.461
2860.59	1250.478
886.8218	3832.715
3593.561	2037.622
1102.306	618.1395
749.3035	899.289
289.7605	546.9103
92.44475	2.061224
1435.521	597.9581
2469.293	365.0782
119.3061	26.85714
480.8614	701.1103
576.4875	966.6399
1830.732	520.8879
360.9209	1525.993
ND	1250.574
ND	4036.647

15.11035
42.42147
234.1563
18.04432
91.43887
58.15297
ND
ND
ND
ND

68.03665
149.1786
20.03344
86.1834
41.48159
ND
ND
ND
ND
ND

1307.772
373.8796
1036.866
934.7636
3940.78
1040.657
ND
ND
ND
ND
